# Intermittent whole-body vibration attenuates a reduction in the number of the capillaries in unloaded rat skeletal muscle

**DOI:** 10.1186/1471-2474-15-315

**Published:** 2014-09-26

**Authors:** Akinori Kaneguchi, Junya Ozawa, Seiichi Kawamata, Tomoyuki Kurose, Kaoru Yamaoka

**Affiliations:** Major in Medical Engineering and Technology, Graduate School of Medical Technology and Health Welfare Science, Hiroshima International University, 555-36 Gakuendai, Kurose, Higashi-Hiroshima, Hiroshima Japan; Department of Rehabilitation, Faculty of Rehabilitation, Hiroshima International University, 555-36 Gakuendai, Kurose, Higashi-Hiroshima, Hiroshima Japan; Graduate School of Biomedical & Health Sciences, Hiroshima University, 1-2-3 Kasumi, Minami-Ku, Hiroshima, Hiroshima Japan

**Keywords:** Hindlimb suspension, Skeletal muscle, Capillary, Angiogenic factor, Muscle atrophy, Whole-body vibration, Weight bearing

## Abstract

**Background:**

Whole-body vibration has been suggested for the prevention of muscle mass loss and muscle wasting as an attractive measure for disuse atrophy. This study examined the effects of daily intermittent whole-body vibration and weight bearing during hindlimb suspension on capillary number and muscle atrophy in rat skeletal muscles.

**Methods:**

Sixty male Wistar rats were randomly divided into four groups: control (CONT), hindlimb suspension (HS), HS + weight bearing (WB), and HS + whole-body vibration (VIB) (n = 15 each). Hindlimb suspension was applied for 2 weeks in HS, HS + WB, and HS + VIB groups. During suspension, rats in HS + VIB group were placed daily on a vibrating whole-body vibration platform for 20 min. In HS + WB group, suspension was interrupted for 20 min/day, allowing weight bearing. Untreated rats were used as controls.

**Results:**

Soleus muscle wet weights and muscle fiber cross-sectional areas (CSA) significantly decreased in HS, HS + WB, and HS + VIB groups compared with CONT group. Both muscle weights and CSA were significantly greater in HS + WB and HS + VIB groups compared with HS group. Capillary numbers (represented by capillary-to-muscle fiber ratio) were significantly smaller in all hindlimb suspension-treated groups compared with CONT group. However, a reduction in capillary number by unloading hindlimbs was partially prevented by whole-body vibration. These findings were supported by examining mRNA for angiogenic-related factors. Expression levels of a pro-angiogenic factor, vascular endothelial growth factor-A mRNA, were significantly lower in all hindlimb suspension-treated groups compared with CONT group. There were no differences among hindlimb suspension-treated groups. Expression levels of an anti-angiogenic factor, CD36 (receptor for thrombospondin-1) mRNA, were significantly higher in all hindlimb suspension-treated groups compared with CONT group. Among the hindlimb suspension-treated groups, expression of CD36 mRNA in HS + VIB group tended to be suppressed (less than half the HS group).

**Conclusions:**

Our results suggest that weight bearing with or without vibration is effective for disuse-derived disturbance by preventing muscle atrophy, and whole-body vibration exercise has an additional benefit of maintaining microcirculation of skeletal muscle.

**Electronic supplementary material:**

The online version of this article (doi:10.1186/1471-2474-15-315) contains supplementary material, which is available to authorized users.

## Background

It is well known that muscle unloading, such as prolonged bed rest [1, 2], spaceflight [3], or rat hindlimb suspension [4], induces muscle atrophy, which is characterized by decreased muscle wet weight and muscle cross-sectional areas (CSA). Concomitant changes in muscle vascularity have been reported, seen as a reduction in capillary number (capillary-to-muscle fiber ratio; C/F ratio) [5–8] and blood vessel diameter [8, 9] in skeletal muscles. Muscle dysfunction in the lower extremities from lack of use (during disuse conditions) could be derived from muscle atrophy [2] as well as impaired blood flow [10].

Avoiding excessive bed rest and maintaining appropriate amounts of daily activity during disuse conditions can suppress the development of muscle atrophy. Other interventions, such as medication [11, 12], electrical stimulation [13], and static or repetitive stretching [14, 15], have been tested to see whether they are capable of counteracting muscle atrophy, and have been shown to be effective to some extent [11–15].

In recent years, short durations of resistive exercise using whole-body vibration has been suggested for the prevention of muscle mass loss and muscle wasting [1, 2], and is considered an attractive countermeasure for disuse atrophy. During whole-body vibration, muscle contractions are evoked via stretch reflexes without voluntary movements in the standing position [16], making whole-body vibration an easy exercise option for frail elderly patients, the poorly motivated, or patients suffering dementia. However, preventive effects of vibration against disuse muscle atrophy is still controversial: vibration on Achilles tendon partially prevented muscle atrophy of unloaded muscles [17], whole-body vibration alone was not as effective at suppressing muscle atrophy as weight bearing [18], or daily intermittent whole-body vibration for 6 weeks caused a reduction in capillary number in mouse soleus muscle [19] .

In this study, whether whole-body vibration with weight bearing are more effective in preventing disuse muscle atrophy than weight bearing alone was examined in the aspects of muscle volume and muscular vascularization using real-time PCR and histological techniques on disuse-induced atrophied muscle. We hypothesized that intermittent whole-body vibration during hindlimb suspension more effectively prevents muscle atrophy than weight bearing alone, but with some disturbance in development of muscular vasucularization.

## Methods

### Animals

Eight-week-old male Wistar rats were used. Rats were randomly divided into four groups (n = 15/group): untreated control (CONT); hindlimb suspension (HS); hindlimb suspension + weight-bearing for 20 min/day (HS + WB); and hindlimb suspension + whole-body vibration for 20 min/day (HS + VIB). All experimental procedures were approved by the committee on animal experimentation of Hiroshima International University.

### Hindlimb suspension

Hindlimb suspension was performed for 2 weeks to induce disuse in HS, HS + WB, and HS + VIB groups according to the method described by Morey-Holton and Globus [20]. Briefly, under anesthesia with an intraperitoneal injection of somnopentyl (0.5 mL/kg body weight), the tail of the rat was loosely wrapped with an adhesive bandage. One end of string was wrapped around the bandage and fixed to the tail, and the other end was attached to a swivel suspended from the top of a cylindrical cage (height 36 cm, diameter 32 cm). Rats were elevated to prevent any contact between the hindlimb and the floor, with the forelimb maintaining contact with the floor and being allowed to move rotationally 360°. Rats in the CONT group were housed in normal cages. Rats were housed on a 12-h light–dark cycle at room temperature (20–25°C) and maintained on a diet of rodent chow and water *ad libitum.*

### Whole-body vibration and weight bearing interventions

From one day after the start of hindlimb suspension, whole-body vibration intervention was applied for 14 days in the HS + VIB group. For whole-body vibration treatment, rats were placed on a whole-body vibration platform (JET-VIBE; YKC, Tokyo, Japan) with all four limbs attached (with weight bearing). Vibration intervention at 55-Hz frequency consisted of four 4-min cycles, with an amplitude of 0.55–1.2 mm. Vibration frequency was set at 55 Hz as a maximal value of the whole-body vibration platform, because lower-limb muscle activities increased depending on the increase of vibration frequency [21, 22]. Only the first cycle on the first day was set at a low frequency (35 Hz) to accustom the rats to vibration. After each 4-min vibration exercise, rats were given a 1-min rest standing on the whole-body vibration platform. In total, HS + VIB rats received 16 min of vibration plus weight bearing and 4 min of weight bearing per day. Total of 20 min of weight bearing time per day may be insufficient according to the previous report [23], but we chose this length to test the synergic effects of concomitant vibration for 16 min.

Weight bearing intervention was applied from one day after the start of hindlimb suspension in the HS + WB group. Intermittent weight bearing intervention was conducted by interrupting the suspension, allowing rats’ fore and hindlimbs to touch the floor for 20 min per day during the 14-day intervention period. Interventions were performed during the light cycle, during which rats were almost inactive.

### Tissue preparation

At the end of the experimental period, body weight was measured, and all rats were killed by exsanguination under anesthesia. The soleus muscles of hindlimbs were immediately removed and weighed. The ratios of soleus muscle wet weight to body weight (relative muscle weight) were calculated and used as an index of muscle atrophy. The right soleus muscles were transversely cut at the mid-belly portion, placed on tragacanth gum jelly on a styrene foam board, and rapidly frozen in isopentane cooled by liquid nitrogen for histological study. The left soleus muscles were immediately minced and immersed in RNAlater reagent (Qiagen, Hilden, Germany) for real-time polymerase chain reaction (PCR) analysis. All samples were stored at -25°C until further examination.

### Histological analysis

Transverse sections (10 μm thick) of soleus muscles were cut with a cryostat microtome at -20°C. Sections were stained with hematoxylin and eosin (HE) for measurement of muscle fiber CSA. HE sections were photographed using a DS-Fi1 digital camera (Nikon, Tokyo, Japan) at × 10 magnification, and CSA of more than 100 muscle fibers in each muscle were manually measured using Image J software (National Institutes of Health, Bethesda, MD, USA).

### Immunohistochemistry

Immunohistochemistry was performed to visualize capillaries in soleus muscles. Sections were air dried, fixed in acetone for 10 min, and rehydrated in 0.01 M phosphate-buffered saline (PBS; pH 7.4) twice for 5 min each time. To quench endogenous peroxidase activity, sections were incubated with methanol containing 3% H_2_O_2_ for 20 min. After two 5-min rinses with PBS, sections were incubated with blocking solution (PBS containing 1% normal horse serum) for 20 min, before being incubated overnight at 4°C with anti-platelet endothelial cell adhesion molecule-1 (PECAM-1) antibody (clone TLD 3A12, 1:250 dilution; Becton Dickinson Bioscience, San Jose, CA, USA). After two 5-min rinses with PBS, sections were incubated with the secondary antibody (horse biotinylated anti-mouse IgG, 1:250 dilution; BA-2001, Vector Laboratories, Burlingame, CA, USA) for 30 min. After two 5-min rinses with PBS, sections were incubated with a streptavidin-biotin complex (1:50 dilution; Elite ABC, Vector Laboratories) for 30 min. After rinsing, immunoreactivity was visualized with a Dako EnVision + kit/HRP (DAB) (Dako Japan, Tokyo, Japan). Sections were then washed with distilled water, dehydrated, and mounted.

### Determination of skeletal muscle vascularity

The capillary-to-muscle fiber (C/F) ratio was determined as a global representation of the capillary supply to skeletal muscle. Immunostained PECAM-1 sections were used to count the number of capillaries. One field of each section was randomly photographed at × 10 magnification. Digital images were displayed on a computer, and the number of muscle fibers and capillaries (determined by elimination of vessels with a lumen diameter >10 μm from all vessels [19]) were manually counted according to the method described by Ichinose *et al.*[24]. Briefly, muscle fibers and capillaries showing their entire boundary within the microscopic field were counted as 1, and fibers and capillaries for which the entire cell boundary was not included within the field were counted as 0.5. The total capillary number per area was divided by the total fiber number per area and expressed as the mean C/F ratio for each muscle. Capillary density (number of capillaries/muscle CSA) was not employed as a structural index of blood/oxygen supply to muscle in this study, because capillary density indices may inappropriately indicate increased vascularity in disused skeletal muscle [25].

### Real-time PCR

#### mRNA isolation

RNAlater reagent-immersed muscles were homogenized in TRIzol reagent (Invitrogen, Grand Island, NY, USA) using a PT-3100 Polytron homogenizer (Kinematica AG, Luzern, Switzerland). Chloroform was then added to each tube and the tubes were vigorously shaken. Samples were centrifuged at 9,600 × *g* for 25 min at 4 °C. The aqueous phase was transferred to fresh tubes, and 70% ethanol was added. Using an RNeasy mini kit (Qiagen), total mRNA was extracted according to the manufacturer’s instructions. The optical density at 260/280 nm was measured to determine the concentration and purity (ratio >1.6). RNA quality was further confirmed by electrophoresis. Samples were supplemented with 1% RNasin ribonuclease inhibitor (Promega, Madison, WI, USA) and stored at -80 °C until further analysis.

#### Reverse transcription

Using total mRNA and the SuperScript III First-strand synthesis system (Invitrogen), cDNA was prepared by reverse transcription according to the manufacturer’s instructions. cDNA samples were stored at -20°C until used for real-time PCR.

#### Real-time PCR for quantifying tissue mRNA

The 7300 Real Time PCR System (Applied Biosystems, Foster City, CA, US) was used to perform TaqMan probe-based real-time PCR reactions. TaqMan primer and probe sets for vascular endothelial growth factor-A (VEGF-A) (Rn00582935_m1), vascular endothelial growth factor-receptor 2 (VEGF-R2) (Rn00564986_m1), transforming growth factor-β1 (TGF-β1) (Rn00572010_m1), thrombospondin-1 (TSP-1) (Rn01513693_m1), CD36 (Rn01442639_m1), and S18 (Rn01428913_gH) were designed and synthesized by Applied Biosystems. S18 was used as an internal control. Amplification reactions contained 10 μL Master mix, 1 μL TaqMan probe, 1 μL cDNA sample, and 8 μL ultrapure water. Amplification was performed using the following thermal cycling profile: initial denaturation at 95°C for 10 min, followed by 40 cycles of 95°C for 15 s, and 60°C for 1 min. If data were not within mean ± 2 standard deviations (SDs), these data were excluded from analysis.

### Statistical analysis

All data are expressed as mean ± SD. For all data, the Shapiro–Wilk test of normality was applied. For body weight, relative muscle weight, and gene expression of VEGF-R2, one-way analysis of variance (ANOVA) and the Tukey’s post-hoc test were applied. For C/F ratio and gene expressions of VEGF-A, TGF-β1, and TSP-1 which assumption of homoscedasticity was not met for data, ANOVA and the Dunnett’s T3 test were alternatively applied. For CSA and gene expression of CD36, the Kruskal–Wallis test was applied, followed by a Mann–Whitney *U* test with Bonferroni adjustment. All statistical analyses were performed using Dr. SPSS II for Windows (SPSS Japan Inc., Tokyo, Japan).

## Results

### Muscle wet weight and muscle fiber CSA

Muscle wet weight-to-body weight (relative muscle weight) ratios of control and unloaded soleus muscles were calculated (Table 1). Compared with the CONT group, the relative weights of soleus muscles in HS, HS + WB, and HS + VIB groups were significantly reduced by 46%, 37%, and 37%, respectively. The relative weights of soleus muscles in the HS + WB and HS + VIB groups were significantly greater than those in the HS group. There was no significant difference between HS + WB and HS + VIB groups.

**Table 1:** **Body weight, relative muscle weight, and muscle fiber CSA**

	CONT	HS	HS + WB	HS + VIB
Body weight (g)	307 ± 8	294 ± 20	277 ± 15*	274 ± 12*
Relative muscle weight (mg/g body weight)	0.32 ± 0.02	0.17 ± 0.02*	0.20 ± 0.02*#	0.20 ± 0.02*#
Muscle fiber CSA (μm^2^)	2529 ± 417	985 ± 213*	1476 ± 238*#	1296 ± 84*#

Values are mean ± standard deviation. *CONT* control, *HS* hindlimb suspension, *HS* + *WB* hindlimb suspension and weight bearing, *HS* + *VIB* hindlimb suspension and intermittent whole-body vibration, *CSA* cross sectional area. *P < 0.05 compared with CONT; # P < 0.05 compared with HS.

**Figure 1:**
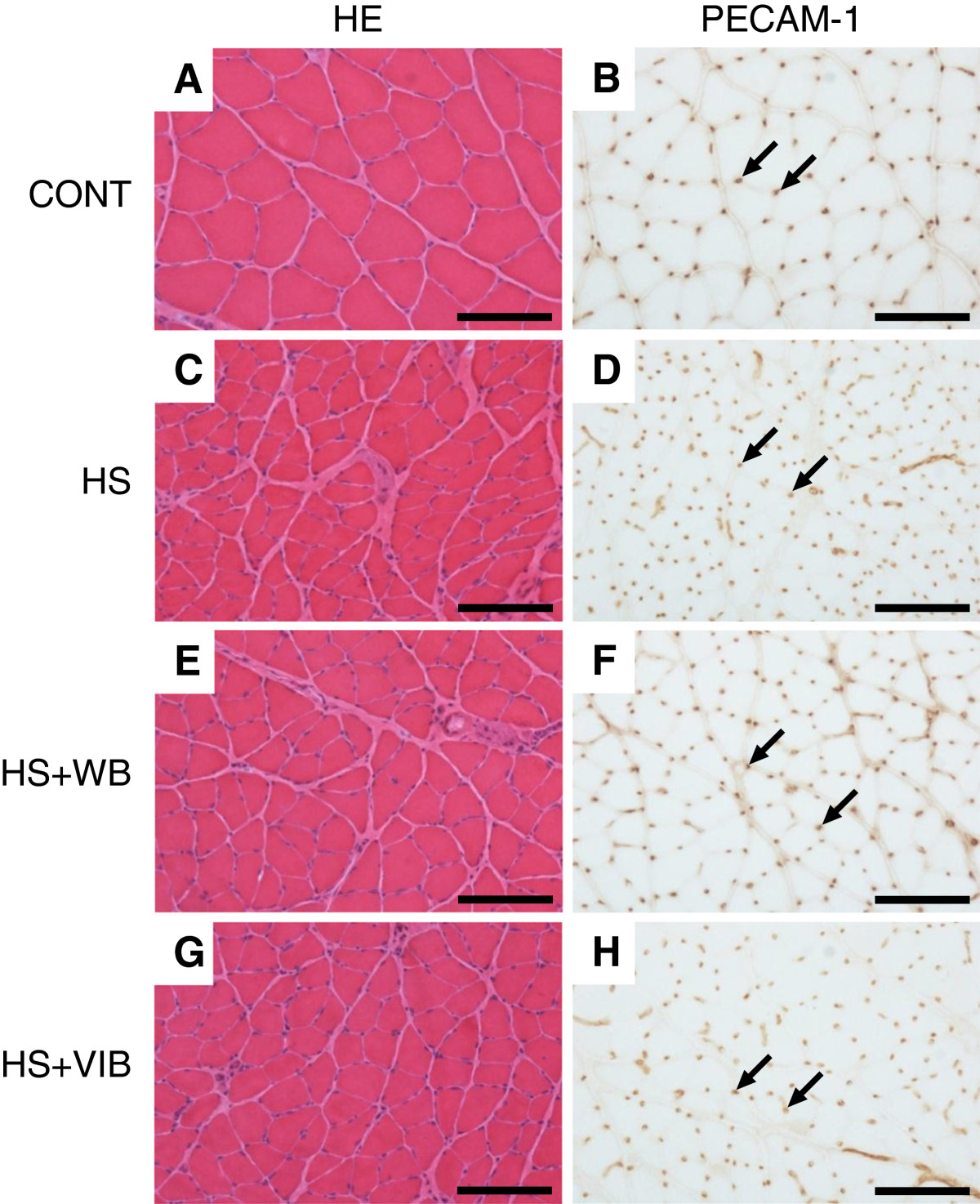
**Representative photographs of soleus muscles. A**, **C**, **E**, and **G** show HE staining. **B**, **D**, **F**, and **H** show immunostained sections for PECAM-1. **A** and **B** show CONT sections; **C** and **D** show HS sections; **E** and **F** show HS + WB sections; and **G** and **H** show HS + VIB sections. In HS **(C)**, marked muscle fiber atrophy was observed. Muscle fiber atrophy was also observed in HS + WB **(E)** and HS + VIB **(G)**, but to a milder extent than that seen in HS. In HS **(D)**, capillary number looked greatest because of marked muscle fiber atrophy, but actually the C/F ratio was the smallest. Scale bar: 100 μm; arrows: capillaries.

Muscle fiber CSA in HS, HS + WB, and HS + VIB groups were significantly smaller than those in the CONT group (39%, 58%, and 51% of CONT, respectively). CSA in HS + WB and HS + VIB groups were significantly greater than those in the HS group. There was no significant difference in CSA between HS + WB and HS + VIB groups (Table 1 and Figure 1).

### Capillary-to-muscle fiber ratio

**Figure 2:**
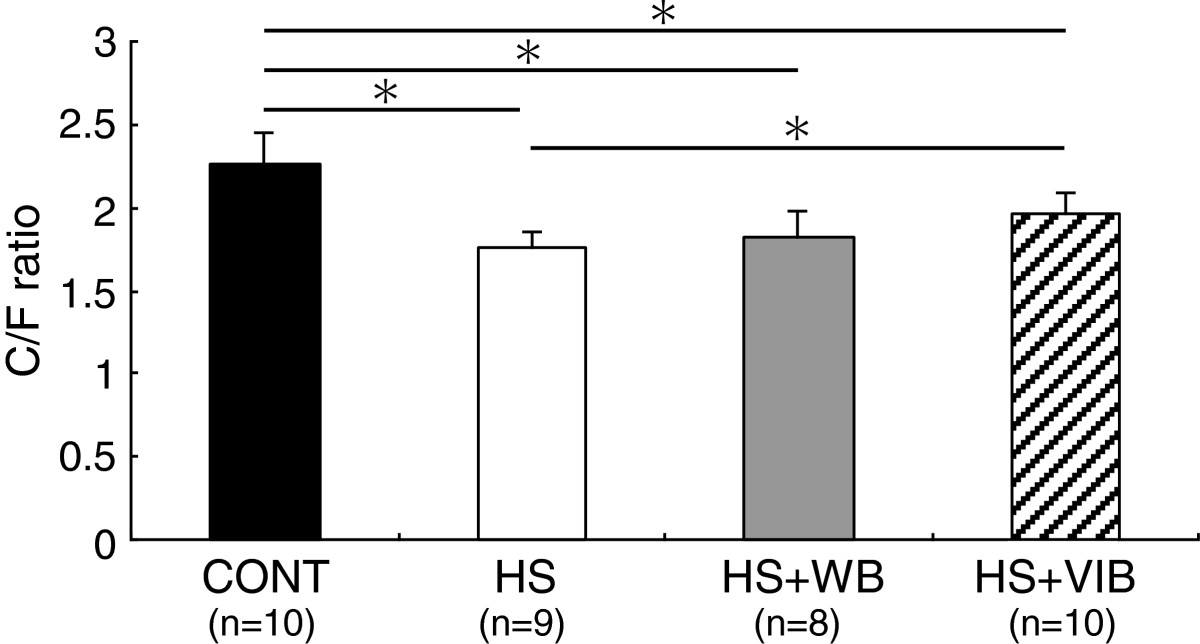
**Capillary number-to-muscle fiber ratio in soleus muscles.** C/F ratios were significantly smaller in HS, HS + WB, and HS + VIB groups compared with the CONT group. However, a reduction in the number of capillaries by hindlimb suspension was partially prevented by whole-body vibration. Values are mean ± SD. *indicates significant differences (P < 0.05).

C/F ratios were significantly smaller in HS (1.75 ± 0.10, 77%), HS + WB (1.83 ± 0.15, 81%), and HS + VIB (1.97 ± 0.13, 87%) groups compared with the CONT group (2.27 ± 0.19). Compared with the HS group, the C/F ratio in the HS + VIB group was significantly greater. There was no significant difference between HS and HS + WB groups (Figures 1 and 2).

### Angiogenic-related factor gene expression

#### Pro-angiogenic factors

**Figure 3:**
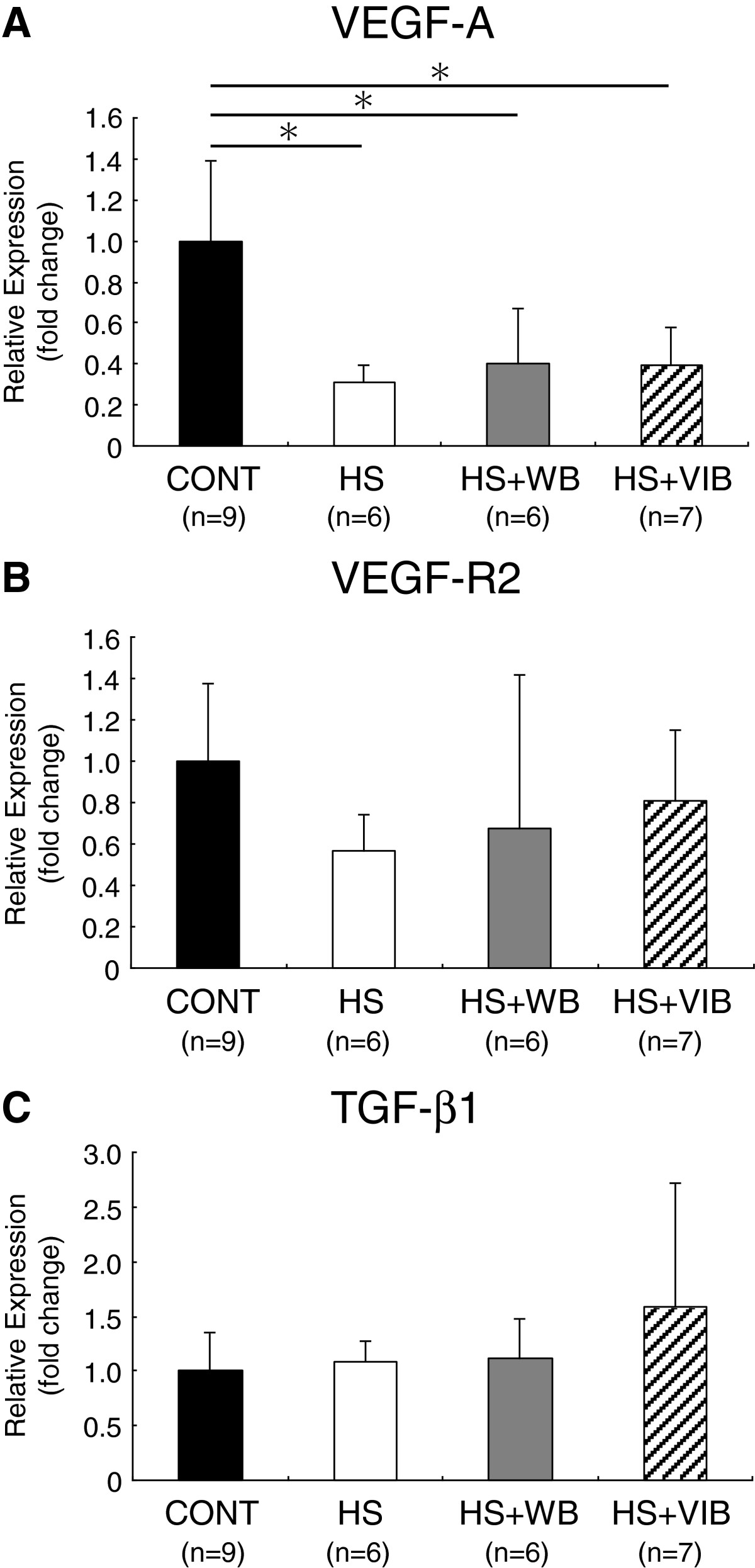
**Gene expression levels of pro-angiogenic factors.** Relative expression of VEGF-A mRNA to S18 ribosomal RNA **(A)**, VEGF-R2 mRNA to S18 ribosomal RNA **(B)**, and TGF-β1 mRNA to S18 ribosomal RNA **(C)** in soleus muscles. Gene expression levels of VEGF-A **(A)** significantly decreased in HS, HS + WB, and HS + VIB groups compared with the CONT group. VEGF-R2 mRNA expression **(B)** in HS group tended to decrease compared with the CONT group, but did not reach statistical significance. There were no significant differences in gene expression levels of TGF-β1 among the groups. Values are mean ± SD. *indicates significant differences (P < 0.05).

Expression levels of VEGF-A mRNA in HS, HS + WB, and HS + VIB groups significantly decreased compared with the CONT group (31%, 40%, and 40% of the CONT, respectively). There were no significant differences in expression levels of VEGF-A mRNA among the HS-treated groups (Figure 3A).Expression levels of VEGF-R2 mRNA in HS, HS + WB, and HS + VIB groups were 56%, 67%, and 81% of the CONT group, respectively. Although VEGF-R2 mRNA expression in the HS group tended to show a decrease compared with the CONT group, there was no significant difference between the groups (Figure 3B).Expression levels of TGF-β1 mRNA in HS, HS + WB, and HS + VIB groups were 108%, 112%, and 159% of the CONT group, respectively. There were no significant differences among the groups (Figure 3C).

#### Anti-angiogenic factors

**Figure 4:**
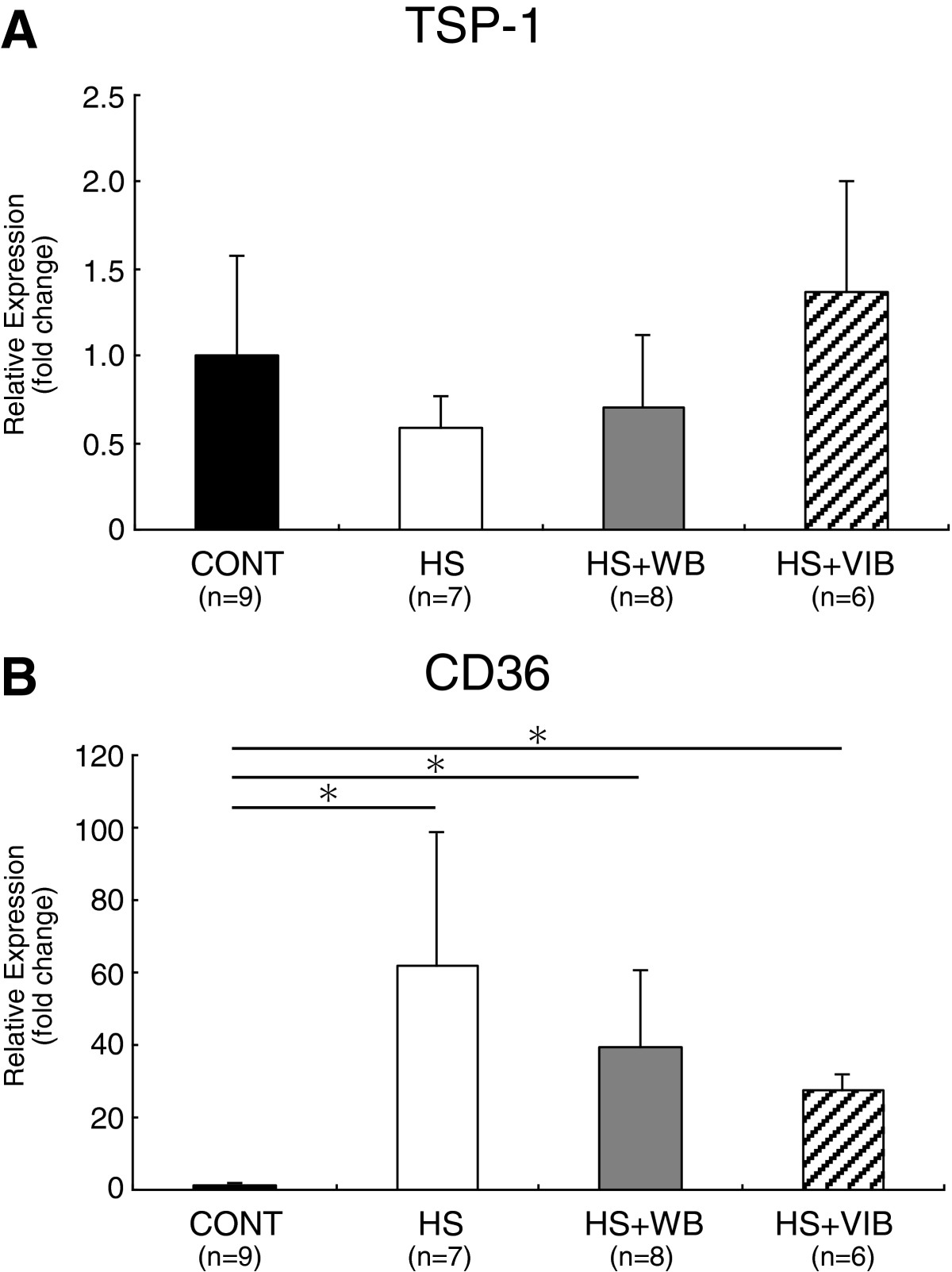
**Gene expression levels of anti-angiogenic factors.** Relative expressions of TSP-1 mRNA to S18 ribosomal RNA **(A)**, and CD36 mRNA to S18 ribosomal RNA **(B)** in soleus muscles. TSP-1 mRNA expression **(A)** in the HS + VIB group tended to increase compared with the HS group, but there were no significant differences between the groups. Compared with the CONT group, gene expression levels of CD36 dramatically increased in HS, HS + WB, and HS + VIB groups. Among the HS-treated groups, although the expression level of CD36 mRNA in the HS + VIB group was less than half that of the HS group, there were no significant differences among HS-treated groups. Values are mean ± SD. *indicates significant differences (P < 0.05).

Expression levels of TSP-1 mRNA were 58%, 70%, and 137% of the CONT group in HS, HS + WB, and HS + VIB groups, respectively. There were no significant differences among the groups, although mean TSP-1 mRNA expression in the HS + VIB group was more than 2-fold that of the HS group (Figure 4A).Compared with the CONT group, the expression levels of CD36 mRNA in HS, HS + WB, and HS + VIB groups dramatically increased (62-, 39-, and 28-fold that of the CONT group, respectively). There were no significant differences among the HS-treated groups. The expression level of CD36 mRNA in the HS + VIB group was less than half the HS group (Figure 4B).

## Discussion

### Capillary reduction by hindlimb suspension

Vascularization adapts to an altered functional demand in skeletal muscle. Exercise increases capillary numbers [26, 27], and disuse condition reduces capillary numbers in human and rat muscles [5–8]. Angiogenesis is known to be stimulated by hypoxia, and stretch and shear stress on vascular endothelial cells in muscle tissue, which are enhanced by exercise [28, 29]. With such stimulation, hypoxia does not appear to occur with hindlimb suspension intervention, because gene expression of hypoxia-inducible factor-1α in muscle was reported not to be altered after 2 weeks of hindlimb suspension [5]. Stretch, an angiogenic stimulation, seems to decrease in hindlimb suspension condition, because soleus muscles shorten in the plantar flexion position in hindlimb suspension [30]. Blood flow, another angiogenic stimulation that induces shear stress in capillaries, was significantly lower in soleus muscle of the hindlimb suspended rat compared with muscle in the quiescent standing state [31]. Therefore, capillary reduction may be a consequence of the absence or a decrease in pro-angiogenic stimuli, such as hypoxia, stretch and shear stress in muscle and capillaries of the hindlimb suspended rat. In the current study, it was demonstrated that angiogenic-relatated factors, such as VEGF-A and CD36 was considered to be involved in capillary reduction in HS group.

Angiogenesis is regulated by a balance between pro- and anti-angiogenic factors [32]. With regard to pro-angiogenic factors, Roudier *et al.*[7] reported that VEGF-A protein levels were unaffected after 9 days of hindlimb suspension. However, Fujino *et al.*[5] reported that both VEGF-A mRNA and VEGF protein levels significantly decreased after 2 weeks of hindlimb suspension. The data of the current study aligned with the findings of Fujino *et al*. With regard to the VEGF receptor, it was reported that VEGF-R2 protein significantly decreased in soleus muscles after 9 days of hindlimb suspension [7]. Our result also showed a tendency for VEGF-R2 mRNA to decrease in HS group, but there was no significant difference in VEGF-R2 mRNA expression between HS and CONT groups. Another pro-angiogenic regulator, TGF-β1 mRNA, examined in this study was unaffected by 2 weeks of hindlimb suspension. This finding is consistent with a previous report that showed no alteration in expression of TGF-β1 protein at 14 days after the hindlimb suspension [33]. Taken together, these results suggest that attenuation of VEGF-A/VEGF-R2 signaling contributed to the reduction in capillary number induced by hindlimb suspension in this study.

TSP-1 protein was reported to increase in unloaded muscles, coinciding with a reduction in capillary number [7]. TSP-1 is generally recognized as an anti-angiogenic factor, and is activated by interaction with the CD36 receptor [34, 35]. Inhibition of endothelial cell proliferation by TSP-1 was completely blocked by the administration of receptor CD36 antibody [36]. Binding of TSP-1 to CD36 induced apoptosis in endothelial cells [35]. Therefore, enhanced TSP-1/CD36 signaling leads to a reduction in capillary number. However, our data showed no significant increase of TSP-1 mRNA in HS group. Conversely, the expression level of CD36 mRNA dramatically increased (62-fold compared with the CONT group) after 2 weeks of hindlimb suspension in this study. To the best of our knowledge, this is the first study to report on the upregulation of CD36 mRNA in disused skeletal muscle. From this study, therefore, it may be suggested that increased TSP-1/CD36 signaling leading to a reduction in capillary distribution was established mainly by CD36 mRNA upregulation but not by TSP-1.

### Effect of whole-body vibration intervention on muscle angiogenesis during hindlimb suspension

Two weeks of hindlimb suspension induced muscle atrophy and significantly reduced capillary numbers in rat muscles. Our results suggest that both daily intermittent weight bearing and whole-body vibration could partially prevent hindlimb suspension-induced muscle atrophy, and capillary numbers were maintained at lower levels in hindlimb suspension-treated groups compared with the CONT group (Table 1 and Figure 2). It should, however, be noted that whole-body vibration partially prevented the development of capillary reduction as the C/F ratio was greater than that of HS group (Figure 2).

The mechanism for the preventive effect on capillary reduction by whole-body vibration is probably suppression of anti-angiogenic factors as well as enhancement of pro-angiogenic factors. The former mechanism may be indicated by the graded decrease in the expression of CD36 mRNA among the hindlimb suspension-treated groups (Figure 4), although the difference was not statistically significant. The latter mechanism seems not to be responsible for whole-body vibration capillary-maintaining effects, because no pro-angiogenic factor was enhanced by whole-body vibration (Figure 3). However, whole-body vibration induced TSP-1 expression by more than 2-fold compared with HS group, although with no statistical significance (Figure 4). An increase in TSP-1 bound to CD36 normally suppresses angiogenesis, yet several studies have demonstrated the pro-angiogenic effects of TSP-1 [37, 38]. The non-binding form of TSP-1 may act as a pro-angiogenic molecule, as the N-terminal domain of TSP-1 has a pro-angiogenic effect [34, 39], which is mediated by α3β1 [40] and α9β1 [39] integrin. TSP-1-stimulated angiogenesis was demonstrated by Bongrazio *et al.*[41], in which shear stress-driven angiogenesis in prazosin-treated mice was accompanied by reduced expression of CD36 protein in endothelial cell fractions and increased expression of TSP-1 protein in whole muscle homogenates. In human, whole-body vibration causes increase in lower-limb blood flow velocity [42, 43] which augments shear stress. Therefore, the same mechanism may be involved in our whole-body vibration-mediated angiogenesis during hindlimb suspension.

In contrast to the current study, a previous study reported that 6 weeks of daily intermittent whole-body vibration induced a reduction in capillary number in mouse soleus muscle [19]. To explain this discrepancy, we propose two possibilities. First, alteration of endothelial cell function by hindlimb suspension [44] might be involved in difference in the sensitivity of lower limb vascularization to whole-body vibration. Second, differences of stimulus condition (e.g., vibration frequency and amplitude) might affect the outcomes of vascularization, because changes in both vibration frequency and amplitude affect human leg blood flow velocity [43]. Therefore, optimal stimulus condition should be investigated in further studies.

### Muscle atrophy

In this study, skeletal muscle atrophy, characterized by a reduction in relative wet weight and CSA, was partially prevented by both intermittent whole-body vibration and weight bearing.

Some pathogenic mechanisms specific to hindlimb suspension may be involved in the muscle atrophy observed in this study. For example, muscle atrophy induced by immobilization is more severe at the shortened muscle position than at the normal length [45]. With hindlimb suspension, the ankle joint is kept at the plantar flexion angle, which makes the soleus muscle shorter [30]. Thus, it seemed that decreases in stretch stimulation in shortened muscle should be one factor of hindlimb suspension-induced atrophy. Consistently, intermittent short-term stretch stimulation during a disuse period was reported to suppress muscle atrophy [14, 15]. During weight bearing, ankle joints were passively dorsiflexed, which provides stretch stimulation to the soleus muscles. Interestingly, muscle atrophy induced by hindlimb suspension could be attenuated solely by contact with the sole of the foot [46, 47]. The mechanism was suggested to be soleus muscle activation mediated by proprioceptive reflex [46].

A previous study indicated that intermittent weight bearing intervention on unloaded hindlimb prevents muscle atrophy in a time-dependent manner [23]. Therefore, prolongation of both whole-body vibration and weight bearing intervention time (>20 min/day) would lead to better outcome. Further studies are necessary to confirm the hypothesis.

Whole-body vibration evokes leg muscle contraction via stretch reflexes [16]. Six weeks of daily whole-body vibration induced soleus muscle hypertrophy in adolescent mice [48]. Therefore, we expected that whole-body vibration would be more effective than weight bearing as a countermeasure for muscle atrophy; the intervention applied for whole-body vibration in this study was actually composed of weight bearing and vibration. However, we could not recognize a synergistic interaction between weight bearing and vibration. This unexpected result may be because of impaired function of muscle spindles under hindlimb suspension conditions [49]. Accordingly, it has been reported that the Achilles tendon reflex was strongly inhibited in muscles of hindlimb suspended rat [50]. Similar observations were made by Zange *et al.*[18], who reported that, compared with weight bearing, whole-body vibration was not sufficient to prevent muscle loss caused by 2 weeks of bed rest.

## Conclusions

We investigated the effects of daily intermittent intervention by short duration weight bearing and whole-body vibration during 2 weeks of hindlimb suspension on capillary distribution and muscle volume in rat soleus muscles. Two weeks of hindlimb suspension caused muscle atrophy and a reduction in capillary number. Both daily weight bearing and whole-body vibration partially prevented muscle atrophy. Importantly, capillary reduction was partially attenuated by whole-body vibration during hindlimb suspension. These results suggest that daily whole-body vibration during disuse conditions may be able to prevent muscle dysfunctions, such as muscle wasting and reduction in fatigue resistance, by partially maintaining both volume and microcirculation of skeletal muscle.

## Authors’ original submitted files for images

Below are the links to the authors’ original submitted files for images. Authors’ original file for figure 1 Authors’ original file for figure 2 Authors’ original file for figure 3 Authors’ original file for figure 4

## Abbreviations

VIB: Whole-body vibration
WB: Weight bearing
CONT: Control
HS: Hindlimb suspension
CSA: Cross-sectional areas
C/F: Capillary to muscle fiber
HE: Hematoxylin and eosin
PBS: Phosphate-buffered saline
PECAM-1: Platelet endothelial cell adhesion molecule-1
VEGF-A: Vascular endothelial growth factor-A
VEGF-R2: Vascular endothelial growth factor-receptor 2
TGF-β1: Transforming growth factor-β1
TSP-1: Thrombospondin-1.

## References

[1] Belavy DL, Miokovic T, Armbrecht G, Rittweger J, Felsenberg D (2009). Resistive vibration exercise reduces lower limb muscle atrophy during 56-day bed-rest. J Musculoskelet Neuronal Interact.

[2] Mulder ER, Horstman AM, Stegeman DF, de Haan A, Belavy DL, Miokovic T, Armbrecht G, Felsenberg D, Gerrits KH (2009). Influence of vibration resistance training on knee extensor and plantar flexor size, strength, and contractile speed characteristics after 60 days of bed rest. J Appl Physiol.

[3] Fitts RH, Riley DR, Widrick JJ (2000). Physiology of a microgravity environment invited review: microgravity and skeletal muscle. J Appl Physiol.

[4] Ohira Y, Nomura T, Kawano F, Sato Y, Ishihara A, Nonaka I (2002). Effects of nine weeks of unloading on neuromuscular activities in adult rats. J Gravit Physiol.

[5] Fujino H, Kohzuki H, Takeda I, Sasai N, Murakami S (2008). Capillary remodeling and inhibition of angiogenic growth factors in disused skeletal muscle (in japanese). Rigakuryoho Kagaku.

[6] Hikida RS, Gollnick PD, Dudley GA, Convertino VA, Buchanan P (1989). Structural and metabolic characteristics of human skeletal muscle following 30 days of simulated microgravity. Aviat Space Environ Med.

[7] Roudier E, Gineste C, Wazna A, Dehghan K, Desplanches D, Birot O (2010). Angio-adaptation in unloaded skeletal muscle: new insights into an early and muscle type-specific dynamic process. J Physiol.

[8] Kano Y, Shimegi S, Takahashi H, Masuda K, Katsuta S (2000). Changes in capillary luminal diameter in rat soleus muscle after hind-limb suspension. Acta Physiol Scand.

[9] Bleeker MW, De Groot PC, Rongen GA, Rittweger J, Felsenberg D, Smits P, Hopman MT (2005). Vascular adaptation to deconditioning and the effect of an exercise countermeasure: results of the Berlin Bed Rest study. J Appl Physiol.

[10] Mulder ER, Kuebler WM, Gerrits KH, Rittweger J, Felsenberg D, Stegeman DF, de Haan A (2007). Knee extensor fatigability after bedrest for 8 weeks with and without countermeasure. Muscle Nerve.

[11] Ricart-Firinga C, Stevens L, Canu MH, Nemirovskaya TL, Mounier Y (2000). Effects of beta(2)-agonist clenbuterol on biochemical and contractile properties of unloaded soleus fibers of rat. Am J Physiol Cell Physiol.

[12] Yamazaki T (2005). Effects of intermittent weight-bearing and clenbuterol on disuse atrophy of rat hindlimb muscles. J Jpn Phys Ther Assoc.

[13] Fujita N, Murakami S, Arakawa T, Miki A, Fujino H (2011). The combined effect of electrical stimulation and resistance isometric contraction on muscle atrophy in rat tibialis anterior muscle. Bosn J Basic Med Sci.

[14] Agata N, Sasai N, Inoue-Miyazu M, Kawakami K, Hayakawa K, Kobayashi K, Sokabe M (2009). Repetitive stretch suppresses denervation-induced atrophy of soleus muscle in rats. Muscle Nerve.

[15] Yamazaki T, Tachino K (2004). Comparison of the effects of intermittent weight bearing and short duration stretching on disuse atrophy of rat soleus muscles. Journal of the Tsuruma Health Sci Med Kanazawa Univ.

[16] Ritzmann R, Kramer A, Gruber M, Gollhofer A, Taube W (2010). EMG activity during whole body vibration: motion artifacts or stretch reflexes?. Eur J Appl Physiol.

[17] Falempin M, In-Albon SF (1999). Influence of brief daily tendon vibration on rat soleus muscle in non-weight-bearing situation. J Appl Physiol.

[18] Zange J, Mester J, Heer M, Kluge G, Liphardt AM (2009). 20-Hz whole body vibration training fails to counteract the decrease in leg muscle volume caused by 14 days of 6 degrees head down tilt bed rest. Eur J Appl Physiol.

[19] Murfee WL, Hammett LA, Evans C, Xie L, Squire M, Rubin C, Judex S, Skalak TC (2005). High-frequency, low-magnitude vibrations suppress the number of blood vessels per muscle fiber in mouse soleus muscle. J Appl Physiol.

[20] Morey-Holton ER, Globus RK (2002). Hindlimb unloading rodent model: technical aspects. J Appl Physiol.

[21] Pollock RD, Woledge RC, Mills KR, Martin FC, Newham DJ (2010). Muscle activity and acceleration during whole body vibration: effect of frequency and amplitude. Clin Biomech (Bristol, Avon).

[22] Krol P, Piecha M, Slomka K, Sobota G, Polak A, Juras G (2011). The effect of whole-body vibration frequency and amplitude on the myoelectric activity of vastus medialis and vastus lateralis. J Sports Sci Med.

[23] Yamazaki T, Nakahira Y, Tachino K (2006). Rat hiramekin no haiyousei isyuku yobou ni oyobosu tanjikan kajuu no kouka (Effects of short duration weight bearing in prevention of disuse muscle atrophy in rat soleus muscle) (in japanese). Rigakuryouhou journal.

[24] Ichinose E, Kurose T, Daitoku D, Kawamata S (2008). The skeletal muscle vascular supply closely correlates with the muscle fiber surface area in the rat. Arch Histol Cytol.

[25] Tyml K, Mathieu-Costello O (2001). Structural and functional changes in the microvasculature of disused skeletal muscle. Front Biosci.

[26] Gavin TP, Ruster RS, Carrithers JA, Zwetsloot KA, Kraus RM, Evans CA, Knapp DJ, Drew JL, McCartney JS, Garry JP, Hickner RC (2007). No difference in the skeletal muscle angiogenic response to aerobic exercise training between young and aged men. J Physiol.

[27] Waters RE, Rotevatn S, Li P, Annex BH, Yan Z (2004). Voluntary running induces fiber type-specific angiogenesis in mouse skeletal muscle. Am J Physiol Cell Physiol.

[28] Egginton S (2009). Invited review: activity-induced angiogenesis. Pflugers Arch.

[29] Prior BM, Yang HT, Terjung RL (2004). What makes vessels grow with exercise training?. J Appl Physiol (1985).

[30] Riley DA, Slocum GR, Bain JL, Sedlak FR, Sowa TE, Mellender JW (1990). Rat hindlimb unloading: soleus histochemistry, ultrastructure, and electromyography. J Appl Physiol.

[31] McDonald KS, Delp MD, Fitts RH (1992). Effect of hindlimb unweighting on tissue blood flow in the rat. J Appl Physiol.

[32] Olfert IM, Birot O (2011). Importance of anti-angiogenic factors in the regulation of skeletal muscle angiogenesis. Microcirculation.

[33] Hirose T, Nakazato K, Song H, Ishii N (2008). TGF-beta1 and TNF-alpha are involved in the transcription of type I collagen alpha2 gene in soleus muscle atrophied by mechanical unloading. J Appl Physiol.

[34] Bornstein P (2009). Thrombospondins function as regulators of angiogenesis. J Cell Commun Signal.

[35] Jimenez B, Volpert OV, Crawford SE, Febbraio M, Silverstein RL, Bouck N (2000). Signals leading to apoptosis-dependent inhibition of neovascularization by thrombospondin-1. Nat Med.

[36] Anderson CR, Hastings NE, Blackman BR, Price RJ (2008). Capillary sprout endothelial cells exhibit a CD36 low phenotype: regulation by shear stress and vascular endothelial growth factor-induced mechanism for attenuating anti-proliferative thrombospondin-1 signaling. Am J Pathol.

[37] Nicosia RF, Tuszynski GP (1994). Matrix-bound thrombospondin promotes angiogenesis in vitro. J Cell Biol.

[38] Taraboletti G, Morbidelli L, Donnini S, Parenti A, Granger HJ, Giavazzi R, Ziche M (2000). The heparin binding 25 kDa fragment of thrombospondin-1 promotes angiogenesis and modulates gelatinase and TIMP-2 production in endothelial cells. FASEB J.

[39] Staniszewska I, Zaveri S, Del Valle L, Oliva I, Rothman VL, Croul SE, Roberts DD, Mosher DF, Tuszynski GP, Marcinkiewicz C (2007). Interaction of alpha9beta1 integrin with thrombospondin-1 promotes angiogenesis. Circ Res.

[40] Chandrasekaran L, He CZ, Al-Barazi H, Krutzsch HC, Iruela-Arispe ML, Roberts DD (2000). Cell contact-dependent activation of alpha3beta1 integrin modulates endothelial cell responses to thrombospondin-1. Mol Biol Cell.

[41] Bongrazio M, Da Silva-Azevedo L, Bergmann EC, Baum O, Hinz B, Pries AR, Zakrzewicz A (2006). Shear stress modulates the expression of thrombospondin-1 and CD36 in endothelial cells in vitro and during shear stress-induced angiogenesis in vivo. Int J Immunopathol Pharmacol.

[42] Kerschan-Schindl K, Grampp S, Henk C, Resch H, Preisinger E, Fialka-Moser V, Imhof H (2001). Whole-body vibration exercise leads to alterations in muscle blood volume. Clin Physiol.

[43] Lythgo N, Eser P, de Groot P, Galea M (2009). Whole-body vibration dosage alters leg blood flow. Clin Physiol Funct Imaging.

[44] Schrage WG, Woodman CR, Laughlin MH (2000). Hindlimb unweighting alters endothelium-dependent vasodilation and ecNOS expression in soleus arterioles. J Appl Physiol (1985).

[45] Jokl P, Konstadt S (1983). The effect of limb immobilization on muscle function and protein composition. Clin Orthop Relat Res.

[46] De-Doncker L, Picquet F, Falempin M (2000). Effects of cutaneous receptor stimulation on muscular atrophy developed in hindlimb unloading condition. J Appl Physiol.

[47] Kyparos A, Feeback DL, Layne CS, Martinez DA, Clarke MS (2005). Mechanical stimulation of the plantar foot surface attenuates soleus muscle atrophy induced by hindlimb unloading in rats. J Appl Physiol.

[48] Xie L, Rubin C, Judex S (2008). Enhancement of the adolescent murine musculoskeletal system using low-level mechanical vibrations. J Appl Physiol (1985).

[49] Tang B, Fan XL, Wu SD (2004). Effects of tail suspension on electrophysiological characteristics of muscle spindles in isolated rat soleus muscle. Space Med Med Eng (Beijing).

[50] Anderson J, Almeida-Silveira MI, Perot C (1999). Reflex and muscular adaptations in rat soleus muscle after hindlimb suspension. J Exp Biol.

[CR51] The pre-publication history for this paper can be accessed here:http://www.biomedcentral.com/1471-2474/15/315/prepub

