# Supplementary figures and images for: Intermittent whole-body vibration attenuates a reduction in the number of the capillaries in unloaded rat skeletal muscle
Source: BMC Musculoskelet Disord. 2014 Sep 26;15:315. doi: 10.1186/1471-2474-15-315 (PMC4189584; doi:10.1186/1471-2474-15-315)

HE

PECAM-1

CONT

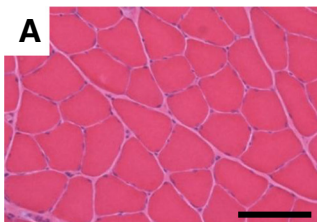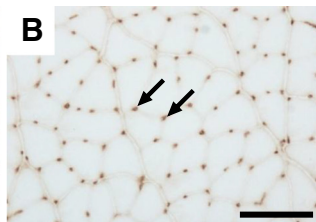

HS

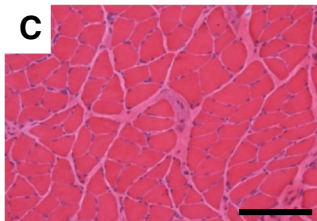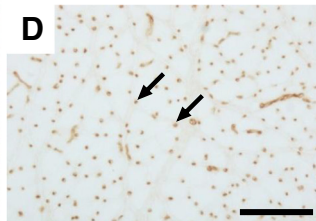

HS+WB

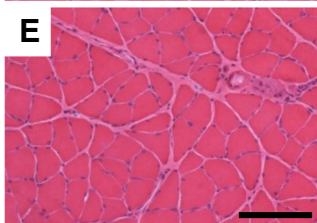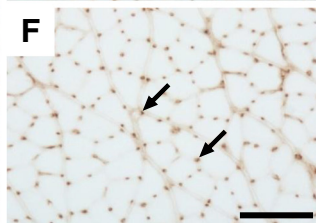

HS+VIB

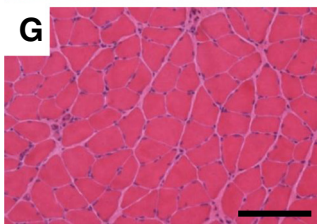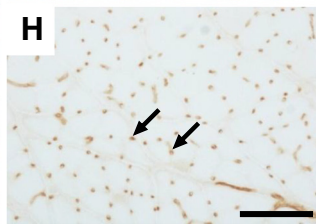

Supplement: Supplementary file 1 — Authors’ original file for figure 1 [file 12891_2014_2251_MOESM1_ESM.pdf]

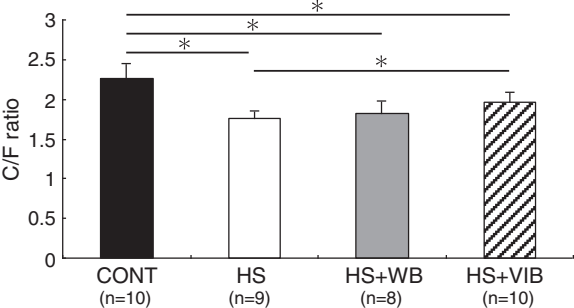

Supplement: Supplementary file 2 — Authors’ original file for figure 2 [file 12891_2014_2251_MOESM2_ESM.pdf]

**A****VEGF-A**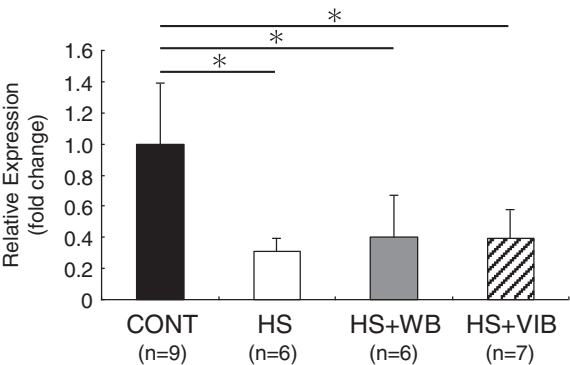**B****VEGF-R2**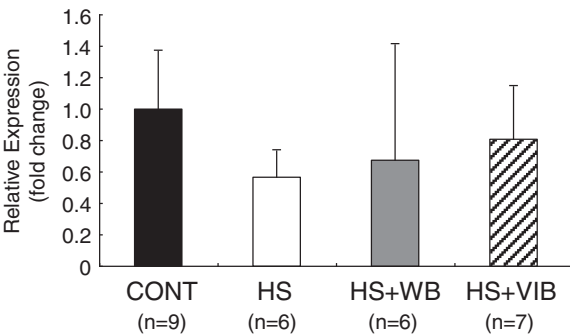**C****TGF- $\beta$ 1**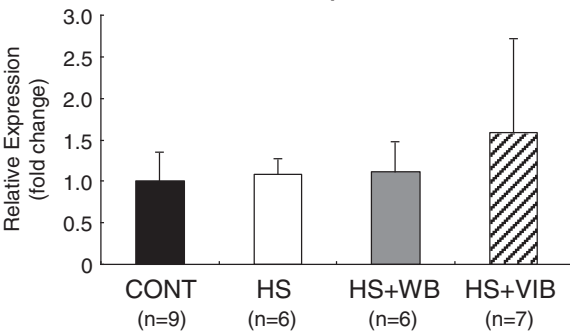

Supplement: Supplementary file 3 — Authors’ original file for figure 3 [file 12891_2014_2251_MOESM3_ESM.pdf]

**A****TSP-1**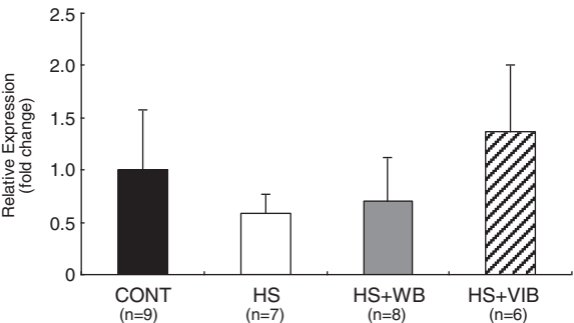**B****CD36**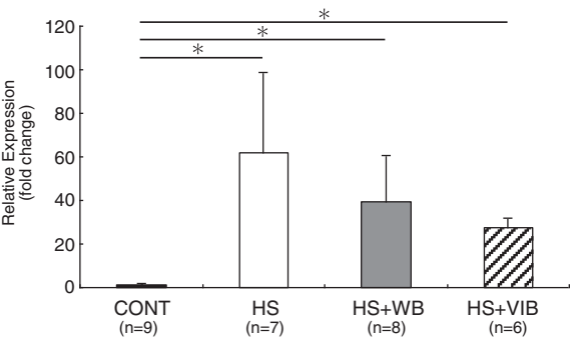

Supplement: Supplementary file 4 — Authors’ original file for figure 4 [file 12891_2014_2251_MOESM4_ESM.pdf]
